# Oxidized LDL Induces Pro‐Inflammatory Transcriptomic and Epigenomic Responses in Human CD4 ^+^ T Cells

**DOI:** 10.1096/fj.202503657R

**Published:** 2026-02-18

**Authors:** Toby A. Brown, Anil Chalisey, Jiahao Jiang, Chris A. O'Callaghan

**Affiliations:** ^1^ Centre for Human Genetics, Nuffield Department of Medicine University of Oxford Oxford UK

**Keywords:** atherosclerosis, epigenomics, helper‐inducer T‐lymphocytes, inflammation, LDL lipoproteins, transcriptome

## Abstract

Elevated circulating low‐density lipoprotein cholesterol (LDL‐C) is a key risk factor for coronary artery disease (CAD). The pathogenesis of CAD is multifactorial, driven by heritable and lifestyle‐related risk factors. Although CD4^+^ T cells are one of the main cell types in atherosclerotic lesions, their interaction with atherogenic oxidized LDL (ox‐LDL) remains poorly understood. Therefore, we sought to characterize the transcriptomic and epigenomic consequences of ox‐LDL on activated human CD4^+^ T cells. We find that ox‐LDL causes a shift towards a pro‐inflammatory, cytokine‐producing CD4^+^ T cell transcriptomic state. Concurrently, ox‐LDL induces genome‐wide changes in chromatin accessibility, notably in promoter regions. By integrating our multiomic data, we identify the NRF1 and SP1 transcription factors as likely mediators of ox‐LDL‐induced changes in gene expression. In contrast, the influence of AP‐1 related factors over CD4^+^ T cell gene expression decreases following ox‐LDL stimulation. We leveraged our multiomic data to investigate the disease relevance of ox‐LDL exposure, by investigating genomic locations where CAD‐associated single nucleotide polymorphisms were found within dynamic ox‐LDL‐regulated accessible chromatin regions. Together, we demonstrate a disease‐relevant role for ox‐LDL in atherogenic conditioning of CD4^+^ T cells. Understanding such cell‐type specific interactions with CAD risk factors may facilitate the development of targeted therapies for CAD.

## Graphical abstract

Oxidized low‐density lipoprotein (ox‐LDL) induces transcription of a pro‐inflammatory cytokine network in CD4^+^ T cells. The transcriptional effects of ox‐LDL are coordinated by the NRF1 and SP1 transcription factors. We show overlap of coronary artery disease (CAD)‐associated loci and ox‐LDL‐regulated accessible chromatin regions, including the rs936395 polymorphism, and propose causal mechanisms for prioritized CAD variants.

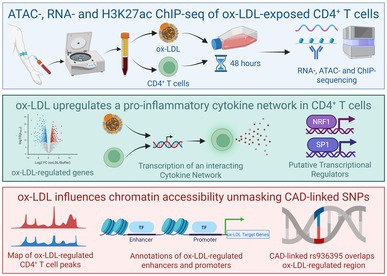

## 1 Introduction

Ischemic heart disease is the leading cause of mortality worldwide [1] and is typically caused by coronary artery disease (CAD). CAD results from atherosclerosis, a chronic inflammatory process causing the formation of plaques that can limit blood flow and trigger occlusive thrombosis [2, 3]. Atherosclerosis is influenced by both genetic and environmental risk factors, which may interact [4], for example, by influencing levels of circulating low‐density lipoprotein (LDL)‐cholesterol, a key correlate of disease risk [3]. LDLs are heterogeneous cholesterol‐carrying particles and are also associated with risk for other complex diseases, including Alzheimer's disease [5] and metabolic syndrome [6]. LDL‐cholesterol is notably pathogenic when oxidized (ox‐LDL) [7]. Ox‐LDL can interact with, and be endocytosed by, immune cells, perturbing their function [8, 9].

CD4^+^ T cells are among the most numerous immune cell types in atherosclerotic lesions accounting for over 30% of plaque‐resident immune cells in single‐cell RNA‐sequencing studies [10]. These plaque CD4^+^ T cells are predominantly activated [10] and exist as different subsets, with distinct functional relevance to atherogenesis [11]. For example, in mouse models, activated T_H_1 and T_H_17 cells promote inflammation in an atherosclerotic context [11, 12], whereas T_reg_ cells are thought to be atheroprotective [13, 14]. CD4^+^ T cells play key roles in the pathogenesis of multiple diseases, including chronic inflammatory and autoimmune diseases [15]. Understanding the interaction between CD4^+^ T cells and the key metabolic risk factor, ox‐LDL, has the potential to cast new light on the pathogenesis of atherosclerosis and other complex diseases associated with LDL‐cholesterol levels.

CD4^+^ T cell differentiation and function are governed by gene expression [11], which is heavily influenced by epigenomic modifications that impact chromatin accessibility [16]. These chromatin changes can influence transcription factor (TF) binding and gene expression. Some TFs act as master regulators, dictating whether CD4^+^ T cells adopt a pro‐inflammatory or atheroprotective transcriptome and phenotype [11, 17].

We sought to characterize how exposure to ox‐LDL modulates activated primary human CD4^+^ T cells, as activated CD4^+^ T cells are exposed to ox‐LDL within atherosclerotic plaques. We reveal a pro‐inflammatory cytokine response to ox‐LDL treatment in which there are epigenomic changes in binding motifs for the nuclear respiratory factor 1 (NRF1) and specificity protein 1 (SP1) TFs. We find that multiple CAD‐associated single nucleotide polymorphisms (SNPs) are located in ox‐LDL‐regulated regions of the genome. We propose possible causal mechanisms for the influence of the rs936395 locus in promoting SP1 binding and the rs1332328 locus in impacting lymphoid lipase A, lysosomal acid type (LIPA) expression. Our genome‐wide assay for transposase‐accessible chromatin (ATAC)‐seq and RNA‐seq data will also provide a useful resource for researchers investigating lymphocytic involvement in other complex diseases, especially those in which ox‐LDL has been implicated, such as Alzheimer's disease.

## 2 Methods

### 2.1 Ethics Statement

Ethical approval for the study was obtained from the NHS Research Ethics Committee (South Central‐Hampshire B, reference 13/SC/0392), and all participants provided informed consent.

### 2.2 Sample Collection & Cell Selection

50 mL of peripheral blood was obtained from healthy volunteers by venesection, and density centrifugation was used to isolate peripheral blood mononuclear cells (PBMCs). PBMCs were then incubated with CD14 microbeads to isolate and remove the blood monocyte population. Unbound cells were incubated with CD4 microbeads prior to activation with bead‐bound anti‐CD3 and anti‐CD28 at 37°C. Ox‐LDL was prepared from fresh human blood as described previously [18, 19, 20]; the properties of the ox‐LDL generated, including the level of oxidation and lack of cellular toxicity, have been reported previously, demonstrating no increase in necrosis or apoptosis [20].

### 2.3 Library Preparation & Sequencing

Following activation, cells were treated for 48 h with 50 μg/mL ox‐LDL or an equivalent volume of control buffer. For RNA‐sequencing, RNA was extracted with TRIzol and the PureLink RNA mini kit (Invitrogen, Waltham, Massachusetts, USA) following the manufacturer's instructions. Illumina (San Diego, CA, USA) reverse‐stranded TruSeq library preparation with polyA selection was performed prior to sequencing. For chromatin immunoprecipitation (ChIP)‐sequencing, the MAGnify Chromatin Immunoprecipitation System (ThermoFisher, Waltham, Massachusetts, USA) was used in accordance with the manufacturer's instructions with 2 μL of the antibody [total rabbit IgG or anti‐H3K27ac (Abcam (Cambridge, UK), ab472)]. Sonicated but untreated lysates were used as an input control. DNA was purified with a Qiagen (Venlo, Netherlands) MinElute kit prior to TruSeq adaptor ligation. For ATAC‐sequencing, samples were repeatedly centrifuged before nuclear pellets were suspended in a transposition reaction mix containing 2.5 μL hyperactive Tn5 transposase from the Illumina DNA sample preparation kit and 22.5 μL nuclease‐free water and incubated for 30 min at 37°C. Transposed DNA was purified with the Qiagen MinElute kit and amplified by PCR. RNA, ATAC, and ChIP libraries underwent 50 base pair (bp) or 75 bp sequencing on Illumina Hi‐Seq 2500 and Hi‐Seq 4000 sequencing platforms.

### 2.4 RNA‐Sequencing Data Analysis

Raw reads were aligned to the human genome GRCh38 with *STAR*, and a count matrix of reads overlapping genes was generated with the *FeatureCounts* package from *Rsubread*. Briefly, downstream differential gene expression analysis was performed with *edgeR* using the quasi‐likelihood F test. Gene set enrichment analysis was performed with *clusterprofiler* for gene ontology (GO) biological process terms. Protein–protein interaction networks were generated from the *STRING* database using *Cytoscape*.

### 2.5 ATAC‐ and ChIP‐Sequencing Data Analysis

ATAC‐sequencing reads were trimmed for adapter content with *NGmerge*, before both ChIP‐ and ATAC‐reads were aligned to the GRCh38 genome with *bowtie2*. Duplicate reads were filtered from ChIP‐ and ATAC‐experiments, and reads aligning to the mitochondrial genome were filtered from ATAC‐sequencing alignments. ATAC‐seq alignments were shifted +4/−5 bp to represent the centre of the Tn5 transposition. Count‐per‐million normalized coverage tracks for both sequencing modalities were generated with *deepTools* and visualized using the *IGV Genome Browser*. Consensus peak sets were generated for ChIP‐ and ATAC‐sequencing using *MACS3*. H3K27ac peaks overlapping the region +/−250 bp of a consensus ATAC peak were deemed to be overlapping. ATAC‐peaks were annotated using *ChIPseeker*, with peaks overlapping a region from −1000 to +200 bp about the transcription start site (TSS) being defined as “promoters”. Differential accessibility analysis for ATAC‐sequencing was performed with *FeatureCounts, csaw*, and *edgeR*. Motif enrichment analysis and TF footprinting were performed on ox‐LDL up‐ and down‐regulated peaks with *HOMER* and *TOBIAS*.

### 2.6 Integration of Transcriptomic and Epigenomic Data

*LISA2* [21] was used to predict transcriptional regulators with an input consensus peak set and a list of differentially expressed genes. This software identifies a series of putative transcriptional regulators and derives *p*‐values for their influence on gene expression. A consensus set of TFs thought to influence ox‐LDL‐regulated gene expression was generated by combining transcriptional regulators predicted by *LISA*, *TOBIAS*, and *HOMER*.

### 2.7 Overlap of Single Nucleotide Polymorphism With Ox‐LDL‐Regulated Genome Locations

Single nucleotide polymorphisms (SNPs), accompanying traits, and *p* values were downloaded from the NHGRI‐EBI Catalog of human variants (accessed January 2024). Only SNPs with significant genome‐wide association (*p* < 5e‐8) and relevant to the search term ‘Coronary artery disease’ were selected for further analysis. These were clumped in Plink (v1.90 beta 7.2) to identify independent risk loci (r^2^ > 0.1) (identified by the Phase 31 000 genomes project 3 in EUR samples, hg38 build); the final set of variants was computed by establishing SNPs in linkage disequilibrium (LD) (r^2^ > 0.8), with those independent risk loci. Common SNP variants (minor allele frequency > 1%, dbSNP155) were used in this study. We then identified SNPs overlapping ox‐LDL‐regulated accessible chromatin regions using the FindOverlaps function within *IRanges*. We prioritized eQTL SNPs, obtained from GTEx v8 (whole blood) and eQTLGen (Blood) databases, for further investigation. To identify motifs disrupted by these SNPs, we used the *R* package *motifbreakR* and the accompanying *hocomoco* motif package (last updated 04/03/22). We selectively identified SNPs interrupting motifs of TFs of interest and plotted relevant SNPs using the plotMB function within *motifbreakR*. These prioritized SNPs were then subject to variant effect prediction and scoring with the deep learning AlphaGenome model for the ontology CL:0000624 (CD4‐positive, alpha‐beta T cell) [22].

### 2.8 Hi‐C Data Analysis

Naïve and activated human CD4^+^ T cell Hi‐C data [23] were downloaded from the Sequence Read Archive (BioProject PRJNA521042). Raw files were aligned to the GRCh38 genome using the *HiCUP* pipeline, using the *bowtie2* aligner. The default maximum and minimum di‐tag lengths of 800 and 150, respectively, were used. Downstream analysis of aligned data was performed using *HOMER* after passing the hicup2homer script on *HiCUP* output directories. We searched for significant intrachromosomal interactions using *HOMER* findHiCInteractionsByChr.pl. (−res 5000, ‐superRes 10 000). Sample interaction files were then merged, and duplicate interactions were filtered by selecting the interaction with the highest logP Value. We then isolated the TSS (−1000 bp +250 bp) from our annotation file and used *HOMER annotateInteractions.pl* to define significant physical interactions between promoter regions and regions with ATAC‐ and H3K27ac‐ChIP‐seq signals (excluding those annotated by *ChIPseeker* as promoters), terming these regions ‘putative enhancers’. Gene ontology enrichment analysis for genes in contact with ox‐LDL‐upregulated (log_2_ (fold change in chromatin accessibility) > 1) ‘putative enhancers’ was performed with *clusterprofiler*.

## 3 Results

### 3.1 Ox‐LDL Initiates an Inflammatory Transcriptomic Response in CD4 ^+^ T Cells

**FIGURE 1:**
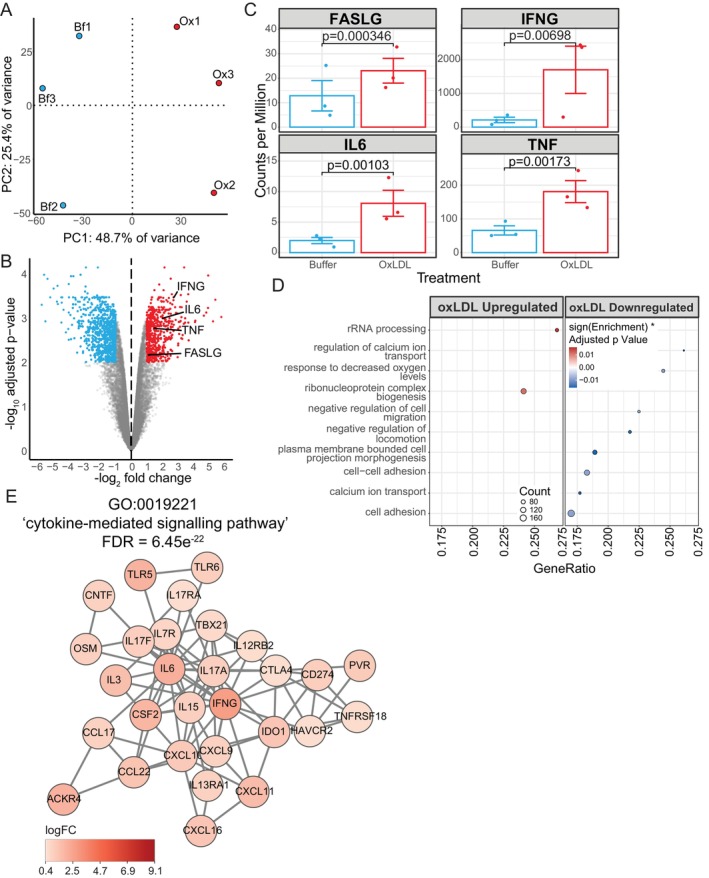
Ox‐LDL triggers pro‐inflammatory changes in gene expression in CD4^+^ T cells. (A) PCA of CD4^+^ T cells from 3 donors treated with ox‐LDL (Ox) or buffer (Bf). The number indicates the donor. (B) Volcano plot of genes expressed in CD4^+^ T cells. Colored dots indicate genes with an FDR < 0.05 and log_2_(fold change) > 1 (red) or < 1 (blue) in cells exposed to ox‐LDL compared to cells exposed to buffer. (C) Plot of read counts per million expressions for selected genes. *p* values were computed using the quasi‐likelihood F‐test, and error bars indicate the mean ± SEM. (D) Gene set enrichment analysis of GO biological process terms for cells exposed to ox‐LDL compared to cells exposed to buffer. (E) PPI network constructed from the *String* database, subclustered with granularity 2.5. Nodes are colored by log_2_ (fold change).

As most CD4^+^ T cells in human atherosclerotic plaques are activated [10], primary human CD4^+^ T cells were isolated from fresh human blood and activated with CD3/CD28 costimulation. They were then exposed to either ox‐LDL or buffer and analyzed using RNA‐sequencing. Principal component analysis (PCA) (Figure 1A) demonstrates that the major source of variance between all the samples (PC1) is attributable to the exposure of the cells to ox‐LDL. In contrast, the distribution of PC2 is consistent with the expected biological variation between individuals. Differential gene expression (DGE) analysis identified a total of 5211 differentially expressed genes (Table S1). Using our previous meta‐analysis of atherosclerotic plaque single‐cell RNA‐sequencing datasets [18], we found a good correlation (*r* = 0.80) between whole transcriptome expression in activated plaque CD4^+^ T cells and our ex vivo CD4^+^ T cells. Exposure to ox‐LDL increased the expression of 853 genes by more than 2‐fold and lowered the expression of 1221 genes by more than 2‐fold compared to cells exposed to buffer (Figure 1B). Upregulated genes included those associated with inflammatory activity, such as TNF, IFNG, FASLG, and IL6, as well as genes involved in metabolism, such as ACOX1. These upregulated cytokines have also been shown to be upregulated by ox‐LDL in monocytes and peripheral blood mononuclear cells [24, 25] (Figure 1C). However, there was no correlation (*r* = 0.01, *p* = 0.74) between the effect size of gene expression changes induced by CD4^+^ T cell activation [26] and ox‐LDL exposure. Previously, we have also shown that ox‐LDL exposure promotes pro‐inflammatory gene expression in primary human macrophages, with notable enrichment of the TNF receptor signaling pathway [19]. Gene set enrichment analysis identified GO biological process terms that were up‐ or down‐regulated (Figure 1D) in CD4^+^ T cells that had been exposed to ox‐LDL. These included changes associated with cellular activation, migration, and cellular accommodation to lower oxygen levels. To identify interacting proteins upregulated by ox‐LDL, we constructed protein networks using the *String* protein–protein interaction (PPI) database. This revealed a large network containing multiple pro‐inflammatory cytokines (Figure 1E).

### 3.2 Ox‐LDL Causes Significant Changes in Open Chromatin Distribution, Increasing Accessibility of SP1 and NRF1 Motifs

**FIGURE 2:**
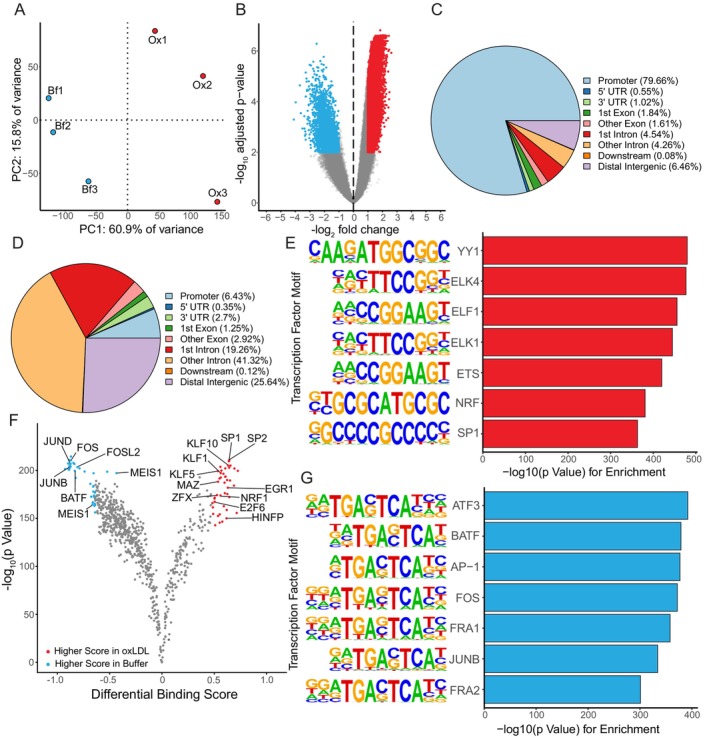
Ox‐LDL causes changes in chromatin accessibility affecting TF binding sites in CD4^+^ T cells. (A) PCA of ATAC‐seq data from CD4^+^ T cells from 3 donors exposed to ox‐LDL (Ox) or buffer (Bf). The number indicates the donor. (B) Volcano plot of chromatin regions with changes in chromatin accessibility triggered by ox‐LDL compared to buffer. Colored chromatin regions have an FDR < 0.05 and log_2_ (fold change in chromatin accessibility) > 1 (red) or < 1 (blue) in ox‐LDL compared to buffer‐treated CD4^+^ T cells. Pie charts of *ChIPseeker* annotations for chromatin regions determined to be more (C) or less (D) accessible following exposure of cells to ox‐LDL. (E) and (G) *HOMER* motif enrichment analysis for chromatin regions that have up‐ (E) or down‐ (G) regulated accessibility induced by exposure of cells to ox‐LDL. Motifs are shown next to their log_10_ (*p* value for motif enrichment over background). (F) Volcano plot of TF footprinting. Dots represent different TFs, and TFs are indicated with colored dots when they were determined as significant by a ‐log_10_ (*p*‐value) above the 95% quantile and/or differential binding scores smaller than the 5% (blue) or larger than the 95% (red) quantiles. TFs are labeled where the TF was determined as a putative regulator by footprinting, motif enrichment, and landscape *in silico* deletion analysis.

Given the substantial changes in gene expression that are triggered by exposure to ox‐LDL, we used ATAC‐seq to characterize the regulatory elements influencing these transcriptomic changes. As with the RNA‐seq data, PCA of the ATAC‐seq data (Figure 2A) demonstrated that the majority of variance between samples was attributable to exposure to ox‐LDL, with the remaining variance consistent with the expected biological variation between individuals. To identify regions of the genome with changes in accessibility induced by ox‐LDL, we performed differential accessibility analysis at consensus open chromatin peaks (Figure 2B). Of the 23 453 differentially accessible peaks identified, 15 166 were more accessible after ox‐LDL exposure and were predominantly located in the promoter regions of genes (Figure 2C) (Table S2). In comparison, genomic regions that were less accessible following ox‐LDL exposure were more frequently in downstream or distal intergenic locations (Figure 2D). We analyzed the frequency of different TF motifs within these chromatin regions that were altered by ox‐LDL relative to the frequency in chromatin regions that were unaffected by ox‐LDL. Notably, motifs for NRF1 (enrichment fold change = 2.32) and SP1 (enrichment fold change = 1.51) were enriched in peaks that were upregulated by ox‐LDL (Figure 2E). Additionally, TF footprinting (Figure 2F) revealed localized regions of lower signals in ATAC‐seq peaks, indicative of TF binding, that overlapped known TF motifs. Among these were the ETS‐domain TFs ELK1 and ELK4 (enrichment fold changes of 1.76 and 1.73, respectively), which have been shown previously to have a role in CD8^+^ T cell differentiation [27]. In contrast, AP‐1 (FOS/JUN)‐related motifs (Figure 2G) were enriched (enrichment fold change = 1.52) in genomic regions where chromatin accessibility was reduced following ox‐LDL exposure, with TF footprinting consistent with a reduction in the presence of TFs bound to these motifs after exposure of CD4^+^ T cells to ox‐LDL (Figure 2F).

### 3.3 NRF1 And SP1 Partly Regulate the Transcriptomic Changes Induced by Ox‐LDL

**FIGURE 3:**
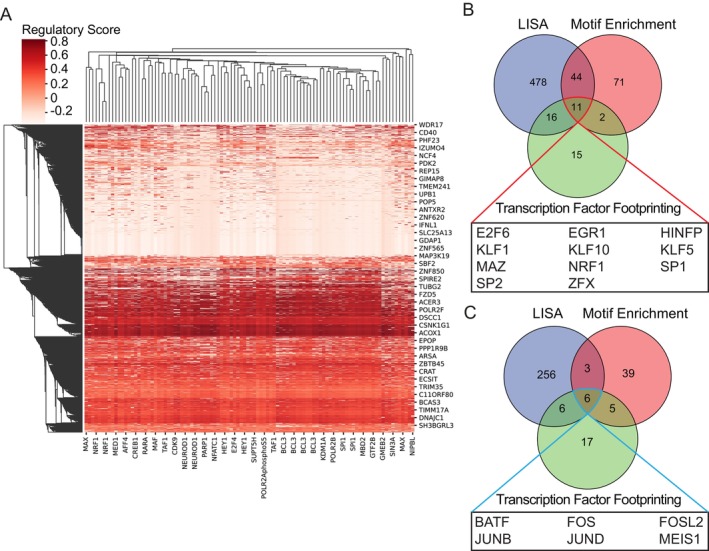
Integrated multiomic analysis demonstrates that exposure of CD4^+^ T cells to ox‐LDL alters TF regulation of gene expression. (A) *LISA* heatmap analysis with regulatory scores. Regulatory scores represent the predicted influence of TFs (x axis) on gene expression (y axis). Higher regulatory scores represent a greater degree of confidence that the TF influences gene expression. (B) and (C) Venn diagrams of TFs predicted by *LISA*, DNA footprinting, and motif enrichment analysis to mediate up‐regulation (B) and down‐regulation (C) of gene expression in response to ox‐LDL. The consensus set of TFs predicted by all 3 approaches is listed below each Venn diagram.

To assess whether ox‐LDL‐induced changes in chromatin accessibility were associated with corresponding changes in gene expression, we sought to integrate our RNA‐ and ATAC‐sequencing data. We applied epigenetic landscape *in silico* deletion analysis (LISA) to identify putative TFs influencing gene expression (Figure 3A). LISA calculates a *p* value and regulatory score for whether a TF that is expressed preferentially influences the expression of a gene over randomly sampled background genes [21], thus predicting dynamically regulated TFs. Among others, NRF1 and SP1 regulatory elements achieve statistical significance for influencing ox‐LDL up‐regulated genes. In contrast, AP‐1 elements are implicated in the downregulation of a set of genes in response to ox‐LDL exposure. LISA heatmap analysis is consistent with the influence of NRF1 on the expression of ACOX1, which is important in fatty acid metabolism (Figure 3A). Integrating our findings from HOMER motif analysis, LISA multiomic integration and TF footprinting enabled us to identify a consensus set of TFs predicted by all three methodologies to regulate ox‐LDL‐induced (Figure 3B) or ‐repressed (Figure 3C) gene expression programs. We applied a further test to these consensus TFs by assessing whether the gene encoding the TF was expressed at a level of > 1 logCPM. Of our 17 consensus TFs, only KLF1 and MEIS1 were not expressed in our human CD4^+^ T cell RNA‐seq dataset. Interestingly, we observed significant downregulation of FOS (log_2_ (fold change ox‐LDL/buffer) = −1.04, FDR = 0.0088) and JUN (log_2_(fold change ox‐LDL/buffer) = −0.722, FDR = 0.024) in response to ox‐LDL, in accordance with the prediction that AP‐1‐related TFs exert less regulatory influence after ox‐LDL exposure.

### 3.4 Ox‐LDL Regulates Chromatin Accessibility at Putative Enhancer Regions

In addition to ATAC‐ and RNA‐sequencing, we also performed H3K27ac‐ChIP‐sequencing on CD4^+^ T cells exposed to ox‐LDL or to buffer to support annotations of accessible chromatin regions [28]. The majority of H3K27ac peaks (Table S3) overlapped with open chromatin regions, primarily in promoter regions (Table S4). However, we also identified 10 224 non‐promoter open chromatin peaks at which there was an H3K27ac signal (Table S4). Using publicly available CD4^+^ T cell Hi‐C data [23], we identified H3K27ac peaks that had significant intrachromosomal contacts with promoter regions, consistent with physical promoter–enhancer interactions [23]. We termed these regions ‘putative enhancers’ and detail the genes whose promoter regions were in contact with each ‘putative enhancer’ (Table S5). The average distance from a putative enhancer region to its promoter contact was 247 kb. Of these putative enhancers, 2126 were at genomic sites where chromatin accessibility was altered by ox‐LDL, consistent with the effect of ox‐LDL on gene regulation being mediated, in part, through dynamic enhancer elements. 1138 of these enhancers had a log_2_(fold change in chromatin accessibility) greater than 1 and the gene set targeted by these enhancers was enriched for genes involved in T cell differentiation (adjusted *p* value = 0.00586). The chromatin conformation capture data reveals that multiple putative enhancers with increased accessibility following ox‐LDL exposure contact the promoter region of one of our pro‐inflammatory differentially expressed genes, TNF (Table S5).

### 3.5 Overlap Between CAD‐Specific SNPs and Ox‐LDL‐Regulated Genomic Sites

**FIGURE 4:**
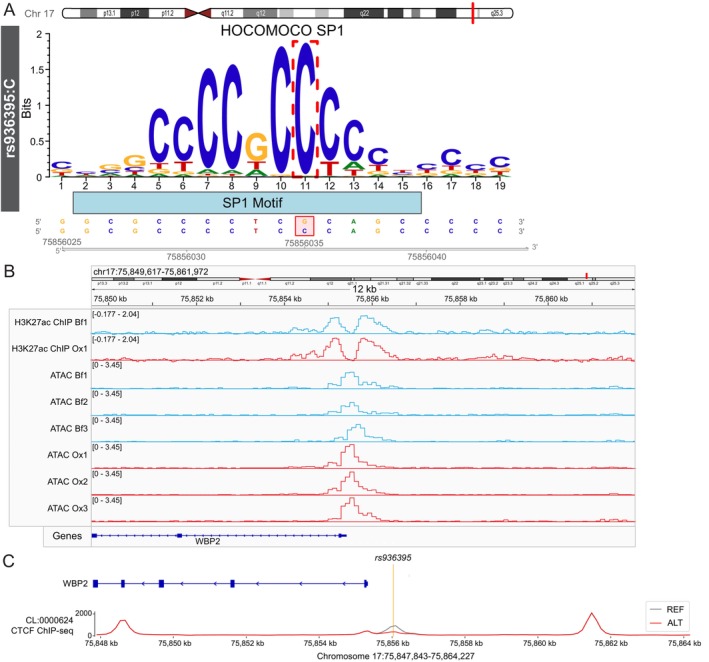
The rs936395 SNP interrupts SP1 and CTCF binding motifs in a region that undergoes a change in chromatin accessibility with oxLDL. (A) The rs936395*:C* SNP increases predicted SP1 binding to an SP1 motif at this location on chromosome 17, plotted by *motifbreakR*. The *p* value threshold was set at 1 × 10^−4^, and motifs were obtained from the HOCOMOCO database. (B) Counts per million‐normalized tracks of both ATAC and input‐normalized ChIP read coverage at the rs936395 locus on chromosome 17. (C) Deep‐learning model prediction of CTCF TF ChIP‐seq tracks on chromosome 17 in CD4^+^ T cells with either the reference ‘G' or alternate ‘C' allele at the rs936395 loci [22].

To assess the clinical relevance of these epigenomic changes, we examined the overlap between disease‐associated SNPs and chromatin regions where accessibility was altered by ox‐LDL. We identified 202 CAD SNPs that were located in genomic regions where chromatin accessibility is up‐ or down‐regulated by exposure to ox‐LDL, 102 of which were identified as eQTLs in either the GTEx v8 or eQTLGen whole blood databases. Notably, 17 of these CAD‐associated eQTL SNPs occurred in TF motifs implicated in the regulation of ox‐LDL‐induced and ‐repressed genes (Table S6). The rs936395 and rs1332328 SNPs were selected for further investigation because of the magnitude of change in chromatin accessibility at these loci and their predicted relevance to lipid biology. The alternate ‘C’ allele at the rs936395 locus increases the predicted binding affinity of SP1 to an SP1 motif at this location (Figure 4A). Closer inspection of ATAC‐sequencing reads indicates that exposure of cells to ox‐LDL is associated with a significant increase in chromatin accessibility at this site (Figure 4B). Interestingly, the AlphaGenome model also predicts that the alternate rs936395 allele may impair CCCTC‐binding factor (CTCF) binding in this region (Figure 4C), perhaps facilitating increased SP1 binding. Separately, we find that the prioritized rs1332328 SNP is an eQTL SNP in whole blood for LIPA (Table S6), a lipid metabolism enzyme [29]. We then used deep‐learning AlphaGenome variant effect scoring to predict the impact of our prioritized variants specifically in CD4^+^ T cells (Table S7). In line with whole blood eQTL data, this analysis also predicts that the alternate rs1332328 'T' allele reduces LIPA expression (raw_score = −0.0360) in human CD4^+^ T cells (Table S7).

## 4 Discussion

CAD is a complex disease involving interactions between multiple cell types and pathways over many decades [10]. In order to dissect this complexity, we have focused on the response of human peripheral blood‐derived CD4^+^T cells to ox‐LDL, as we have done previously with human macrophages and with human endothelial cells [18, 19, 30]. Understanding the specific response of a given cell type to ox‐LDL has the potential to provide important insights into pathophysiology and therapeutic targets. We demonstrate that ox‐LDL triggers substantial changes in gene expression and chromatin accessibility in activated primary human CD4^+^ T cells. The changes include increased expression of a range of pro‐inflammatory genes, including multiple cytokines, and are consistent with the known role of ox‐LDL in the induction of the T_H1_ cell subtype [31]. Among the cytokines upregulated in response to ox‐LDL in activated CD4^+^ T cells are IL6, IFNG, and TNF. These cytokines have all been shown to be upregulated by ox‐LDL in other PBMC‐derived cells [24, 25] and to contribute to the pathophysiology of CAD [32]. A recent study [33] of cellular indexing of transcriptomes and epitopes (CITE)‐sequencing data [10] also revealed upregulation of these cytokine pathways in plaque CD4^+^ T cells, relative to peripheral blood CD4^+^ T cells. These findings suggest that future mechanistic investigation of the interaction between ox‐LDL and CD4^+^ T cell cytokine production and release is warranted. The mechanism whereby ox‐LDL triggers these effects in CD4^+^ T cells is unclear. Ox‐LDL has been reported to act through the LOX‐1 receptor [34] and CD69 [35, 36] in T lymphocytes. A previous transcriptomic study in T cells found that ox‐LDL exposure upregulated expression of the anti‐inflammatory TFs NR4A1 and NR4A3 [36]; although, we did not observe upregulation of these TFs in our study.

We and others have shown that ox‐LDL induces widespread changes in chromatin accessibility in macrophages [18, 19, 25] and in endothelial cells [30] and their progenitors [37]. However, the epigenomic changes triggered by ox‐LDL in human CD4^+^ T cells have not been studied previously. We demonstrate substantial ox‐LDL‐induced changes with notably increased accessibility at promoter regions. The mechanisms by which these promoter‐dominated changes mediate their full modulatory effects in CD4^+^ T cells that have already been activated by CD3/CD28 costimulation remain to be explored in future studies. Mechanisms mediating these changes could involve reactive oxygen species (ROS) [38] or direct activation of histone deacetylases through ERK1/2 signaling [39].

We have identified sites across the genome where exposure of activated CD4^+^ T cells to ox‐LDL alters chromatin accessibility; DNA footprinting and motif analysis identified TFs that are likely to bind to these sites. Notably, NRF1 and SP1 motifs were enriched in ox‐LDL‐regulated chromatin regions. *In silico* deletion analysis also independently inferred a regulatory role for NRF1 and SP1 based on our multiomic data as well as a database of previous ChIP‐seq studies. NRF1 acts in concert with NRF2 to activate expression of various metabolic genes, including those involved in the clearance of cholesterol in the liver [40]. Ox‐LDL has previously been reported to induce the binding of NRF1 to its motifs in macrophages [41]. Similarly, SP1 has been shown to be activated by ox‐LDL in smooth muscle [42] and mesangial cells [43]. These TFs may act synergistically in response to ox‐LDL to stimulate cytokine expression, and future studies could explore this experimentally using approaches including genetic manipulation of the expression of these TFs.

In contrast, there is a reduction in Activator protein 1 (AP‐1) motif activity, indicating a reduction in the influence of AP‐1 TFs following exposure to ox‐LDL in activated CD4^+^ T cells. AP‐1 TFs are generally heterodimers of FOS and JUN proteins that regulate the expression of a wide range of genes. FOS gene expression is downregulated by ox‐LDL, but a TF can also alter the expression of genes without there being a change in the expression of the TF itself [44]. Interestingly, this contrasts with findings in other cell types. Both single‐cell RNA sequencing [45] and immunohistochemistry [46] have shown upregulation of activated AP‐1 in vascular cells and monocytes from atherosclerotic lesions. However, there is also evidence from macrophages to show downregulation of AP‐1 binding at specific promoters in response to ox‐LDL [47]. We have also recently demonstrated activation of AP‐1 sites in primary human macrophages [18] and endothelial cells in response to ox‐LDL [30]. Although AP‐1 typically regulates T cell activation and cytokine production [48], it does not seem to make a net contribution to the pro‐inflammatory cytokine expression we observe in CD4^+^ T cells. This indicates that ox‐LDL can modulate the AP‐1 response in pre‐activated CD4^+^ T cells and that TFs are impacted by ox‐LDL in a cell‐ and genomic region‐specific manner.

Much of CAD heritability is attributed to SNPs that confer increased susceptibility to CAD [4]. We found that numerous CAD‐associated SNPs reside in genomic regions where ox‐LDL alters DNA accessibility. To prioritize these SNPs, we selected those which were eQTL SNPs for ox‐LDL‐upregulated genes. This list was further refined by focusing on SNPs that alter TF motifs predicted to influence the CD4^+^ T cell response to ox‐LDL. This analysis prioritized rs936395, located in either an intronic or promoter region of the WBP2 gene depending on isoform, and rs1332328 near the TSS of the LIPA gene. Notably, the alternate allele of the rs936395 SNP was predicted to reduce the binding of CTCF, which is important for genome organization, while increasing the predicted binding of SP1. Interestingly, neither rs936395 nor rs1332328 has been identified previously as a lead variant but instead are in high LD with the CAD‐associated rs78532451 (r^2^ = 0.936) [49] and rs1412444 (r^2^ = 0.944) [50] SNPs, respectively. The role of the WBP2 gene remains poorly characterized, although recent evidence suggests that WBP2 may act as an oncogene [51]. However, in our RNA‐sequencing dataset, we note that WBP2 is upregulated by oxLDL in our CD4^+^ T cells (log_2_FC = 0.502, *p* = 0.013), suggesting that the function of WBP2 may be impacted by common metabolic risk factors. In contrast, the association of LIPA and CAD is well documented [29, 50]. However, much of the functional characterization of LIPA variants has been performed in myeloid cells [29]. Our finding that the rs1332328 SNP occurs in a genomic region where oxLDL increases chromatin accessibility in CD4^+^ T cells suggests that LIPA also contributes to CAD through an undefined pathway in lymphoid cells. However, the use of deep learning models for variant effect prediction remains nascent [22] and the predictions made by AlphaGenome warrant further experimental validation. Future functional studies, such as CRISPR‐based genome editing or luciferase reporter assays, could be used to investigate the potential causal mechanisms involving these non‐coding variants in CD4^+^ T cells.

In summary, we have characterized the previously undefined CD4^+^ T cell response to ox‐LDL at both the transcriptomic and epigenomic level. We hypothesize that this pro‐inflammatory response is regulated by NRF1 and SP1 TFs and promotes atherogenesis. We identify CAD‐associated SNPs as priority SNPs for investigation in CD4^+^ T cells and present putative causal mechanisms for the rs936395 and rs1332328 loci. Our work illustrates the importance of understanding the cell‐type specific response to pro‐atherogenic risk factors.

## Author Contributions

C.A.O.C. conceived the project. T.A.B., J.J., and A.C. undertook analysis and experimental work. T.A.B. and C.A.O.C. reviewed data and analyses and wrote the manuscript. All authors edited and approved the manuscript.

## Funding

This work was supported by Wellcome Trust (WT), 102290/Z/13/Z. British Heart Foundation (BHF), FS/4yPhD/F/23/34201.

## Conflicts of Interest

The authors declare no conflicts of interest.

## Supporting information


**Data S1:** fsb271571‐sup‐0001‐Data S1.xlsx.

## Data Availability

Raw and processed data are available from the GEO database with accession number GSE298362 (https://www.ncbi.nlm.nih.gov/geo/query/acc.cgi?acc=GSE298362). All packages were used in accordance with the developer's instructions. Custom functions for defining SNP overlaps with epigenomic data can be found at https://github.com/jhjiang2020/multiome_paper.
