# Supplementary material for: Oxidized LDL Induces Pro‐Inflammatory Transcriptomic and Epigenomic Responses in Human CD4 + T Cells
Source: FASEB J. 2026 Feb 18;40(4):e71571. doi: 10.1096/fj.202503657R (PMC12916081; doi:10.1096/fj.202503657R)
Supplement: Supplementary file 1 — Data S1: fsb271571‐sup‐0001‐Data S1.xlsx. [file FSB2-40-e71571-s001.zip › fsb271571-sup-0002-Supinfo02.docx]

| **Supplementary Table** | **Title** | **Legend** |
| --- | --- | --- |
| Supplementary Table 1 | Differentially Expressed Genes | Gene symbols and ENTREZ IDs of differentially expressed genes in oxLDL vs buffer-treated samples as defined by a Benjamini-Hochberg-adjusted p value <0.05 from the *edgeR* QLF test. Log fold changes of gene expression are presented with positive scores indicating increased expression in oxLDL-treated samples. |
| Supplementary Table 2 | Differentially Accessible ATAC Peaks | Genomic locations and ChIPseeker annotations of differentially accessible peaks in oxLDL vs buffer-treated samples, with positive log fold changes representing increased accessibility in oxLDL. P values computed using *edgeR* QLF test and Benjamini-Hochberg adjusted, sorted by genomic location. |
| Supplementary Table 3 | H3K27ac Peaks | Genomic coordinates of H3K27ac peaks called by *MACS3*, sorted by location. |
| Supplementary Table 4 | H3K27ac Overlap with ATAC Peaks | Numbers of H3K27ac peaks overlapping with ATAC peaks. |
| Supplementary Table 5 | Putative Enhancer Regions | Genomic coordinates and contacts of H3K27ac- and ATAC-peaks that have significant interactions with promoter regions - termed 'putative enhancers', sorted by genomic location. |
| Supplementary Table 6 | Prioritised CAD-associated Variants | rsIDs and genomic loci of prioritised CAD variants - with their TF motifs disrupted and eQTL genes. |
| Supplementary Table 7 | AlphaGenome predictions of prioritised variants | Predicted variant effect scores for CAD-prioritised variants for prioritised variants across the following modalities: RNA-seq, ATAC-seq, CHIP_TF, CHIP_Histone. Variant effect scores are presented as raw and quantile scores. Predictions are ranked by most negative to most positive raw score, with negative values representing a reduction in expression/accessibility/binding with the alternate allele. |
